# Everybody's Page

**Published:** 1888-06-23

**Authors:** 


### Everybody's Page.

The Aylesbury Dairy Company, whose average milk yields 13 per cent. of solids, retain an analyst constantly in their service, and have inspectors going about taking milk as it is distributed to the customers. In this way they know exactly what is being supplied, the distributors are deterred from adulterating, and a high standard is maintained.

Chossat found that when an animal loses two-fifths of its weight, or 40 per cent. of its body weight, death took place, always accompanied or preceded by a great lowering of temperature. In his table, you will observe the striking acts are that of the total quantity of fat in the animal's body, 93 per cent. disappears. Other important organs suffer loss also to a large extent; while another very striking fact is, that the nervous system loses only 2 per cent.

One dose of tea in the twenty-four hours is quite sufficient, and many people who are at present troubled with headaches and many of the so-called nervous diseases, would be far better if they never drank tea at all. Especially should all avoid that very great mistake known as high tea. Tea and meat should never be taken together, at least as forming the principal meal. The tannin, an important constituent of the tea, prevents the digestion of the meat.

There can be little doubt that were the bath with friction of the skin regularly employed by those up in years—and the habit, if acquired, can be easily carried on—much suffering and disablement from bronchitis or winter coughs might be avoided, as well as many of those troublesome forms of skin disease so often met with. As a means for preventing colds the bath is only of use if employed frequently, daily if that can be, if not, then as often as possible, and with regularity.

Nitre is sometimes used to take away the turnip taste from milk. A case was tried some time ago in the west of Scotland, where nitre was supposed to have been added to milk. The dairyman pleaded not guilty to using nitre, but said he gave his cows rock salt. The magistrate found the man not guilty of using nitre, but guilty of adulterating his milk, inasmuch as by giving cows rock salt they were thirsty and drank more water, and thus the milk was diluted by an extra quantity of water.

There is a popular impression that while it may be dangerous to meddle with drugs derived from minerals, no harm can come of indulging in remedies of vegetable origin. This is rather a vast generalisation in face of the fact that such poisons as opium, digitalis, strychnine, and strophanthus, are all obtained from plants. That popular tonic, quinine, which the unlearned do not hesitate to prescribe to themselves and their friends for every languid feeling or slight headache, is by no means so safe as is generally believed; and indeed, arsenic alone, of all minerals employed in therapeutics, can compare in danger with the substances we have named.

The popular idea, and too prevalent practice, of taking a glass of spirits to keep out the cold is a great and dangerous delusion. It does the very reverse. The immediate effect no doubt is to impart a sort of warm glow and exhilaration, but this is a feeling and not a reality. The nervous system is abnormally stimulated, and the action of the heart excited, but these are speedily followed by depression. Again, the bloodvessels of the skin are fuller of blood, and in consequence more heat is radiated away from the surface, while the contractile power already referred to as residing in the skin is also impaired, and in these various ways cold is able to exert its hurtful action, while the means for resisting it are greatly lessened by the alcohol.

Here, at least, among many harsh ones, is a good word for tea. Dr. Underwood, Customs Medical Officer at Kirakiang, China, says that the comparative immunity of the Chinese in that region from typhoid fever, although most of the factors favouring it are present in abundance, may be attributed to the fact that cold, unboiled water is rarely or never used when tea can be had.

A new hypnotic, to be commercially known as sulphonal, has been invented. Its proper name, written in full, is "diethylsulphondimethylmethan." If the short name had not been invented we don't think there would have been a rush on the chemists' shops to obtain it; but "diethyl, &c., might have supplanted the old "Peter Piper" alliteration as a lingual test.

A large fortune awaits the man who will devise a remedy which will keep flies from worrying the horse in summer, and at the same time not kill the horse. Carbolic acid washes, the aromatic oils, especially pennyroyal and various other drugs, to say nothing of mechanical appliances, have all been used, but with only temporary benefit. The oil of bay is said to be particularly efficient, and to be extensively used in Switzerland and the South of France.

It is stated in *La Voz de Hipocrates* that in the City of Mexico, containing about 350,000 inhabitants, the quack practitioners, male and female, are more than twenty-three times as numerous as the qualified men, of whom about 400 only have been recognised by the faculty of medicine. The irregular practitioners are thus classified: 1,800 so-called homœopathic doctors, 200 dosimetric doctors, 3,000 male herbalists, 1,000 female herbalists, 100 female dosimetricians, 8,000 compounders, and 300 male and female sorcerers.

Dr. Lauder Brunton has employed strychnine in insomnia from "over-tiredness" with very happy effects. He says that it occurred to him that as strychnine is a powerful—perhaps the most powerful—nervous stimulant that we possess, a small dose of it might have the effect of bringing the depressed nervous system up from the condition of over-fatigue to simple fatigue, and thus inducing sleep. He found that it acted exactly in the manner he expected, and induced comfortable, healthy sleep without any disagreeable effects next day. Dr. Brunton thinks, however, that it is very doubtful whether strychnine would answer in other cases of sleeplessness than those arising from overwork or worry.

Another counterblast to tobacco, and this time a most serious one, comes from Dr. C. S. Jaffresen, the senior surgeon at the Newcastle Eye Hospital, who blames the baneful weed for being the cause of a disease he names *toxic amblyopia*, which, beginning with colour-blindness, in the course of a few months develops into an entire loss of sight. This is alarming enough in itself, but the doctor will not leave the tobacco-smoker any consolation whatever, such as the reflection that he has smoked for years and his sight has never been affected. "The danger," says he, "is the result of progressive tobacco-poisoning, appears suddenly and without warning, and there is no chance for the patients unless they completely drop the use of tobacco. Nor is moderation of any avail, for the poisonous constituents of tobacco vary so greatly amongst different individuals that no quantitative definition of moderation is possible." The doctors are really dealing too severely with poor human nature. No tea for the women, no tobacco for the men! If they had their way we should have no redeeming vices left.

